# The Assembling and Contraction Mechanisms of Striated Muscles

**DOI:** 10.3389/fchem.2018.00570

**Published:** 2018-11-30

**Authors:** Alberto Ciferri, Alvin L. Crumbliss

**Affiliations:** Chemistry Department, Duke University, Durham, NC, United States

**Keywords:** striated muscle, self-assembly, actin, myosin, titin, sarcomers

## Abstract

A novel approach to the description of the assembly mechanism of functional biological structures is presented. The approach is based on the identification of fundamental self-assembling processes to which an additional structurization “engineered” by Nature to optimize functions is superimposed. Application of the approach to the structure and contraction of the striated muscle evidences a key role of the residual liquid crystallinity of a constrained structure and the alteration of the compatibility between the thin and thick filaments driven by ionic interactions. ATP hydrolysis boosts the relaxation process. A strong protein scaffold, engineered during the evolutionary process and based on the selective anchoring of coordinated filaments, directs a demixing tendency of the two filaments toward a sliding motion along the fiber axis. The Huxley-Hanson sliding filament hypothesis aimed to explain the contraction-relaxation function of the striated muscle, but does not offer any clue on the overall assembling mechanism of the myofibril.

## Introduction

The assembly processes of all structures, molecular, or macroscopic ones, must conform to the criteria of shape and chemical compatibility of the building blocks. The former arises from the geometrical shape, and the second from chemical or supramolecular interactions between the components. These two criteria are the basis of current self-assembling theories and are often referred to as shape and chemical recognition or, alternatively, as hard and soft interactions (Ciferri, 2002, 2005; Lehn, 2002).

A recent article has highlighted the translation of molecular order to the macroscopic level for several classes of synthetic and natural macrostructures (Ciferri, 2016a). It was concluded that fundamental self-assembling treatments, such as molecular and supramolecular liquid crystallinity (Flory, 1984; Khokhlov and Semenov, 1985; Khokhlov, 1991; Odijk, 1996; Ciferri, 2004) and supramolecular polymerization (van der Schoot, 2005), describe the occurrence of macroscopically-ordered model polymers. In the case of biological systems, the contribution of basic assembling mechanisms was readily evidenced in relatively simple cases. For instance, in the case of the collagen fibrillogenesis, the geometrical parameters of the growing filaments promoted linear assembly driven by liquid crystallinity (Ciferri, 2016a). Nevertheless, even in such a simple case, the fiber structure included modifications “engineered” by Nature for specific functions of collagen fibers. When particularly thin fibers are needed (for instance collagen II that reinforces the vitreous structure of the eye), lateral growth is blocked by decoration of the fiber lateral surface with collagen type IX during some stage of the polymerization (Ciferri, 2016a).

The objective of the present work is to identify the basic self-assembling mechanism and associated engineered modifications used by Nature to produce even more complex functional systems. Striated muscles, particularly the cardiac one, exhibit a static and a dynamic function. For the past 50 years, the dynamic function of the contraction has been interpreted by the Huxley-Hanson sliding filament model (Hanson and Huxley, 1953; Szent-Györgyi, 2004; Huxley, 2014). This model is based on a structural analysis by X ray diffraction and does not account for recent developments regarding the phase behavior of rigid polymers and ion-polyelectrolyte interaction.

## Striated muscles and the huxley-hanson model

**Figure 1:**
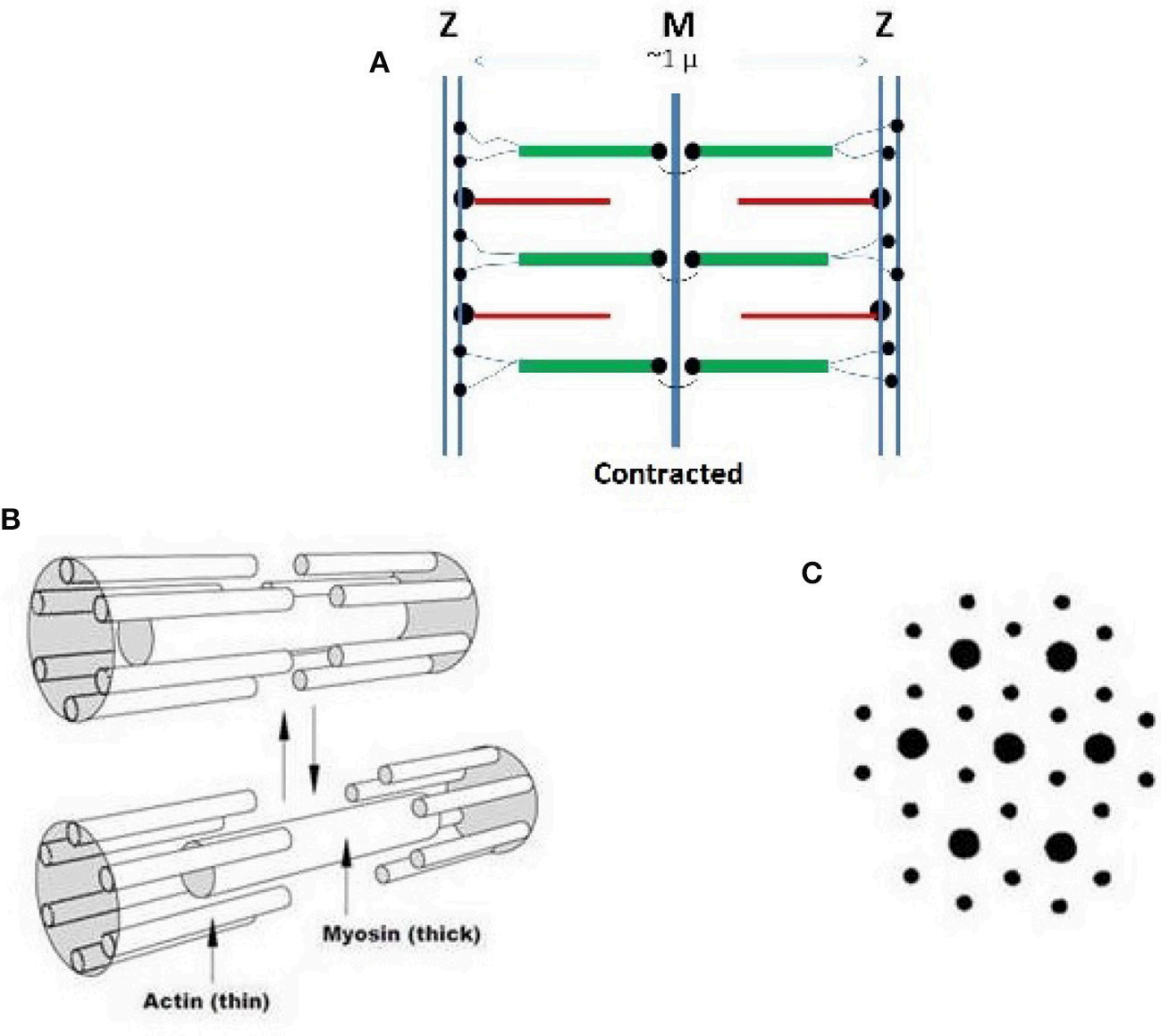
**(A)** Schematization of the structure of a sarcomer in the contracted state based on high resolution optical microscopy. The Z lines limit the length of each repeating sarcomer along a myofibril. The thin filaments are based on rigid actin molecules with bound tropomyosin and troponin. Thin filaments are anchored to the Z region by the cross-linking protein alfa-actinin. The thick filaments) are firmly anchored to the central M zone and are based on rigid myosin bound to three to six tinin molecules. The N-terminals of flexible segments of titin connect each thick filament to the Z region, from which subsequent sarcomeric units depart. The terminal C-sections of titin stabilize opposite half-sarcomers overlaps in the central M zone. Note that thin filaments are polarized and myosin chains with opposite orientation must associate within the two half of a sarcomer. Dots indicate anchoring chemical bond. **(B)** Contraction-relaxation behavior of the smallest conceivable sarcometer that has six connected thin filaments around a thick one. The contracted state is favored by calcium ions released in the sarcometer. Reduction of ionic strength, favors a relaxation of the contraction. **(C)** Electron micrographs of a wider, contracted sarcometer reveal a hexagonal organization of the two types of filaments. Copyright 2014 Creative Common Attribution License.

Striated muscles have a relatively simple morphology with no bends or curvatures but exhibit large complexity in the organization of their main molecular constituents: *actin, myosin, titin*, and numerous minor components, all of which form the contractile *sarcomer* cell schematized in Figure 1A in the contracted state. Linear assembly of sarcomers produce the myofibril that further aggregate into myofibers, (Cooper, 2000; Gregorio and Antin, 2000a,b). Titin, bound to myosin in the thick filament, is a giant protein that might include up to 33,000 amino acids, with an extended length exceeding 1 micrometer that spans the length of the sarcometer (Lee et al., 2007; Hsin et al., 2011). Its tertiary structure is characterized by 244 partially structured domains connected by unstructured sequences. Titin elasticity assists the recovery of the rest length following muscular contraction. It has been interpreted in terms of entropic contributions of the unstructured sequences, and the unfolding of the structured domains (Lee et al., 2007; Hsin et al., 2011).

Thick and thin filaments within the sarcomer interdigitate in a hexagonal patterns where each thick filament is surrounded by six thin ones (Figures 1B,C) (Cooper, 2000; Gregorio and Antin, 2000a,b). During contraction, calcium ions travel from the sarcoplasmic reticulum to the sarcomeric cell following signals from motor neurons (Ca^++^ concentration increases from ca. 10^−7^ to 10^−5^ M). Consistently with the *sliding filament model* originally proposed by Hanson, a weak interaction, ambiguously defined as “salt bridges” links the thin and the tick filaments. This binding allows the thin filament to be pushed toward the center of the sarcomer, causing its shortening and muscle contraction. During relaxation, the salt bridge is dissociated and calcium ions are pushed back to the reticulum.

Recognizing the weakness of the salt bridges (Bosshard et al., 2004), Huxley contributed the idea of a direct link between the thick and the thin filament. His *swinging cross-bridge model* includes ATP hydrolysis, which displaces calcium ions and induces conformational changes in the myosin head of the thick filament. According to his model, calcium ions expose binding sites on troponin and tropomyosin (linked to actin in the thin filament), promoting their binding to the myosin heads. Binding of consecutive molecules of ATP assists the sliding of myosin by “grabbing and swiveling” along the thin filament. In the absence of calcium ions, a change in the configuration of troponin and tropomyosin inhibits the binding of the myosin heads, and relaxation occurs. Luther and Squire have discussed finer details regarding how the rotation of the myosin-titin filaments around their long axes influences the interaction with neighboring actin filaments (Luther and Squire, 2014).

## Analysis of self-assembling contributions

Two main self-assembling features emerge from the appreciation of the chemical composition of the constituent polymers and their spatial orientation. The first remarkable feature is a *soft interaction* contribution due to the polyelectrolyte character of the constituent polymers (Pearstone et al., 1976; Raszkowski et al., 1977; Manning, 1978; Stone and Smillie, 1978; Maruyama et al., 1981; Yang and Janmey, 1996; Xian et al., 1999). The data of Raszkowski et al show that the anionic charge grossly outweighs the cationic one (for myosin: Glu+Asp = 227, Lys+Arg+Hist = 130; for actin: Glu+Asp = 110, Lys+Arg+His = 65 gmol/10^5^ g protein) (Raszkowski et al., 1977). A similar conclusion is obtained from the sequence studies for tropomiosin (Stone and Smillie, 1978) and troponin (Pearstone et al., 1976) (that bind to actin), and titin (Maruyama et al., 1981) (that binds to myosin). These studies reveal that the sarcomeric polymers are all weak polyanions, and therefore programmed to be mutually repulsive unless the fixed anionic charges are connected by the hypothetical “salt bridge,” as claimed by the sliding filament model, or screened by interaction with mobile ions (Manning, 1978; Yang and Janmey, 1996; Xian et al., 1999; Ciferri and Perico, 2010; Ciferri, 2012a; Crumbliss and Parker Siburt, 2012; Maglio et al., 2012). The latter possibility has not been previously considered, but should not be neglected. In fact, even though the Debye screening length is rather small in a 10^−5^ M solution of a conventional electrolyte, calcium ions have a specific binding power that greatly reinforces the simple Debye screening (Ciferri and Perico, 2010; Ciferri, 2012a; Maglio et al., 2012). It should moreover be remarked that current polyelectrolyte theory predicts the occurrence of “condensed” bivalent cations that are free to diffuse along the chain axis but unable to diffuse away (Manning, 1978; Yang and Janmey, 1996; Xian et al., 1999). In addition to a reduced electrostatic repulsion between fixed charges, the ion condensation actually promotes an attractive interaction between chains sharing counter-ions. Condensation effects are predicted to occur above a critical value of charge density, defined by the ratio of the Bjerrum length to the linear charge spacing. The charge density calculated for F-actin in the presence of magnesium ions exceeded the critical value, suggesting significant condensation effects. A contribution to the stabilization of the interdigitated state of the present system is anticipated, but its quantitative evaluation would be problematic.

The second remarkable feature is the *hard interaction* contribution evidenced by the ordered hexagonal distribution of thick and thin filament in the contracted state, which is suggestive of a liquid crystalline assembly (Flory, 1984; Khokhlov and Semenov, 1985; Khokhlov, 1991; Odijk, 1996; Ciferri, 2004). Liquid crystalline structures evolving into smectic, hexagonal mesophases are predicted and observed for solutions of rigid polymers having persistence length above ~50 Å, or axial ratio X (length/diameter) as small as ~3 (Flory, 1984). The reduction of excluded volume within the ordered configuration is the driving force for the thermodynamic stabilization of the mesophase at a critical concentration, *v*^*c*^, inversely related to the axial ratio of the mesogen (Khokhlov, 1991):

*v*^*c*^ ~*3/X* (1).

The lattice treatment evidences that the formation of an ordered mesophase will occur with rigid molecules, as well as with macroscopic rods (Flory, 1984). In the case of the muscle filaments, growth may radially occur within each sarcomer. Actin has one of the largest persistent lengths so far reported, exceeding 1 micrometer (Isambert et al., 1995). However, there are not sufficient data to assess the persistence length of the complexes of interest here. Nevertheless, the axial ratios of the thin and thick segments, which can be tentatively estimated from the average length of the sarcometer (Figure 1A) and the filament diameters [7 and 15 nm, respectively (Cooper, 2000; Gregorio and Antin, 2000a,b)], appear to be in the order of 30. Therefore, in terms of Equation (1), an ordered mesophase will *necessarily develop* in solutions above v ~0,10 of either the actin or myosin complexes (total protein content in the sarcomer is in the order of 20%).

Their mixtures will also produce a *single* mesophase since rigid polymers are predicted to be mutually compatible in ternary solutions displaying athermal or negligible soft interaction between the two solutes (when the Flory-Huggins parameter for the free energy of dilution χ_2, 3_ is close to zero) (Flory, 1984). Alternatively stated, shape compatibility drives the mixing of two mesogens in the absence of any attractive or repulsive soft interaction. A single mesophase is commonly observed with mixtures of low molecular weight liquid crystals when the mesophase is stabilized by *anisotropic*, attractive soft interactions. Flexible segments, occurring within a prevalently rigid molecule, are excluded from the mesophase (Flory, 1984; Khokhlov and Semenov, 1985; Khokhlov, 1991). This situation is consistent with to the disordered elastic sections of titin outside the overlap region.

The above analysis pertains to the case of contracted muscles, when electrostatic repulsion is screened out by calcium ions. Liquid crystalline polyelectrolytes should instead be relevant to the case of the relaxed muscle. Although more detailed experimental or simulation evidence is desirable, the occurrence of the net anionic charge is expected to introduce *repulsion* among the rods, distortions and destabilization of the ordered mesophase. Twisting effects have been described for solutions of a single polymer or polymer mixtures (Nyrkova and Khokhlov, 1986; Odijk, 1996; Ciferri, 2004). Studies on ternary mixtures of rigid polymers displaying soft repulsive interactions (hence poor chemical compatibility and χ_2, 3_ parameters > 0) were reported in the literature of the 1980s. Most systems exhibited demixing into two liquid crystalline phases having different order parameters (Marsano et al., 1984; Sasaki et al., 1988; Maissa and Sixou, 1989; Ciferri, 1991). Therefore, *It is postulated here that a demixing tendency of two anionic polymers is manifested when their fixed anionic charges are unscreened*. A direct verification of the above postulate for the systems considered here would offer additional support to our model. More recent data indicate that compositional segregation effects are also triggered by the presence of a cylindrical body with perpendicular anchoring (Rahini, 2017).

## Engineering the sarcomers

The foregoing analysis applies to solutions of the *unrestricted* actin and myosin components. In the sarcomer, however, opposite half units are strongly bound, and the relative orientation of myosin and actin filaments remains the same on both halves of the sarcomer. *Anchoring* of the filaments appears to be the primary “engineered” strategy used by Nature to produce a one-dimensional motion based on the interpenetration of the two set of filaments. The most interesting strategy is the anchoring of the thick filaments to the M zone, resulting in the interpenetration of the thin rods from two opposite directions, thus magnifying the contraction-relaxation dimensional change.

Another fundamental strategy is the anchoring of several thin segments to the Z zone (Figure 1B) so that a fix spacing between the filaments is assured, a coordinated displacement of several filaments is achieved, and the contraction-relaxation motion involves the whole sarcometer. The latter situation parallels the case of liquid crystals anchored to a surface, which have been extensively investigated due to their great technological relevance (Marrucci and Ciferri, 1977; Ciferri, 2012b). Anchoring results in a further restriction to the lateral displacement of the first layer of anchored molecules.

The net results of the above combined strategies is that *only the motion along the fibrillar axis is allowed*.

The combined role of the calcium ions and of the anchorage is better appreciated by considering that the typical morphology of an unrestricted liquid crystal is based on multi-domain regions (size ca 1 micrometer) that have similar order parameter, but random director orientation (Marrucci and Ciferri, 1977; Ciferri, 2012b). Under these conditions, the postulated demixing would not be coordinated along any specific direction. The engineered anchorage of the filaments within the sarcomer reproduces instead the situation of a *single* liquid crystal and allows a coordinated dimensional change restricted to the myofibril axis (Marrucci and Ciferri, 1977; Ciferri, 2012b).

The early stage of myofibrillogenesis have been documented by electron microscopy in embryonic material (Cooper, 2000; Gregorio and Antin, 2000a,b). During the earliest growth stage, the protein components are randomly dispersed within the cytoplasm. Thereafter, precursors of the Z zone appear, accumulate along the membrane and form complexes with actin and alfa-actinin. During the following stage, complexes of myosin-titin are formed and become localized along the M line. These thick filaments assume their final orientation *before* the thin ones. These observations were considered insufficient to explain the selective localization of the components. The occurrence of a template was indeed postulated (Cooper, 2000; Gregorio and Antin, 2000a,b). However, the above observations are consistent with a primary liquid crystalline orientation of the thick segments anchored along the fibrillar axis, followed by the interdigitation of thin segments that become firmly anchored to the membrane, consistently with an hexagonal topology.

The role of ATP in reducing the polymer-salt interaction is that of a booster of the relaxation process in both models. Additional support for the booster role of ATP comes from the development of the extreme rigidity (*rigor mortis*) manifested by striated muscles when vital functions cease (Jerôme, 1991). It is known that this situation is accompanied by an increase of the concentration of calcium ions in the cytosol, promoted by deterioration of the sarcoplasmatic reticulum and compounded by the absence of ATP, which no longer contributes to the rupture of calcium bridges^1^.

The difference between our model and the Huxley-Hanson one is particularly evident in the case of the contraction step. In the latter model, the stabilization of the contracted state is based on the perplexing hypothesis that myosin filaments make discrete steps on actin, or to hypothetical salt bridges. In our model, the main driving force for the contracted state is the basic *entropic* contribution that drives the athermal mixing of two shape-compatible mesogens within a single liquid crystalline phase (Flory, 1984; Khokhlov and Semenov, 1985; Khokhlov, 1991), possibly enhanced by the parallelizing effect of ion condensation (Manning, 1978; Yang and Janmey, 1996; Xian et al., 1999). In both models, the relaxed configuration is promoted by the removal of calcium ions, but our model emphasizes the prevailing of an *enthalpic* contribution arising from the *r*epulsion of the anionic charges.

There is only partial conjectural evidence for the swinging cross bridge model, which is a however a useful concept in cell motility (Mitchinson and Cramer, 1996). On the other hand, there is compelling evidence for the role of liquid crystalline order on the organization of functional strucures (Marsano et al., 1984; Sasaki et al., 1988; Maissa and Sixou, 1989; Ciferri, 1991, 2016a,b).

It is therefore possible to suggest that the sarcomer retains a memory of the interdigitated liquid crystalline distribution of the unrestricted components formed during the earlier development stages. When the fixed charges on both filaments are screened out (contracted state), full interdigitation is allowed, in line with theoretical predictions for mixtures of two compatible rods (Flory, 1984; Khokhlov and Semenov, 1985; Khokhlov, 1991). However, when the ionic strength of calcium ions is reduced (relaxed state), the increased electrostatic repulsion of fixed charges, coupled with the engineered anchorage of the filaments, will trigger demixing and the linear expulsion of the coordinated thin filaments from the interdigitated configuration (Marsano et al., 1984; Sasaki et al., 1988; Maissa and Sixou, 1989; Ciferri, 1991).

It needs to be pointed out that in addition to a new interpretation of muscular contraction, our model allows an interpretation of the sarcomeric structure based on the superimposition of supramolecular assembling mechanisms to engineered anchorages. Similar approaches might allow the elucidation of even more complex assembly mechanisms of other functional biostructures. Moreover, new guidelines are now suggested to the material scientists attempting to mimic the functions of natural systems.

## Author contributions

AC originated the idea of separating fundamental and engineered assembly mechanisms. ALC offered substantial support and contribution to the organization of the work with input on complexity theory and ionic interaction.

### Conflict of interest statement

The authors declare that the research was conducted in the absence of any commercial or financial relationships that could be construed as a potential conflict of interest.

## Footnotes

^1^Rigor mortis. Wikipedia
